# Early Recognition of Loneliness and Frailty in Relation to Chronic Disease Self-Management: A Quantitative Cross-Sectional Study

**DOI:** 10.3390/healthcare13030266

**Published:** 2025-01-29

**Authors:** Mateja Lorber, Nataša Mlinar Reljić, Sergej Kmetec, Barbara Kegl

**Affiliations:** 1 Faculty of Health Sciences, University of Maribor, 2000 Maribor, Slovenia; natasa.mlinar@um.si (N.M.R.); sergej.kmetec1@um.si (S.K.); barbara.kegl@um.si (B.K.); 2 National Institute of Public Health, 1000 Ljubljana, Slovenia

**Keywords:** self-management, loneliness, frailty, mental health

## Abstract

Background: Chronic disease significantly influences mental health, identity, and self-esteem. It is deeply interconnected with loneliness, frailty, stress, mental health, and the ageing process, forming a complex and interrelated dynamic. The aim was to find an association between loneliness, frailty, mental health, and the patient’s self-management. Methods: A cross-sectional study was conducted between October 2023 and May 2024. A total of 605 patients with chronic disease took part in the research, of whom 67% were female and 33% were male. In total, 71% of participating patients lived in a home environment, and 19% lived in retirement homes. Results: 605 respondents with chronic disease participated in the study and were recruited using a purposive sampling method. Participants were drawn from healthcare settings, including primary care centres, outpatient clinics, and nursing homes, to increase representativeness. The study achieved a response rate of 55% after distributing 1100 questionnaires. Data were analysed with SPSS Statistics 25.0 using descriptive and inferential statistical methods, including non-parametric tests (Kruskal–Wallis test, Mann–Whitney U test) and Spearman’s correlation. The main results showed that patients who self-rated their chronic disease as well- or very well-managed (81%) were less frail (*p* < 0.001), less lonely (*p* < 0.001), and had better mental health (*p* = 0.015). Significant associations were found between frailty, loneliness (*r_s_* = 0.428, *p* < 0.001), and lower mental health (*r_s_* = 0.185, *p* < 0.001). In addition, frequent social contact was associated with lower frailty and loneliness (*p* < 0.001). Conclusions: Without adequate assessment and support from the healthcare system, patients may face challenges in meeting their needs, which can contribute to loneliness, frailty, and mental health decline. It is crucial to acknowledge that every individual with a chronic disease, regardless of age, education level, or condition, must actively participate in managing their chronic disease. Recognising the importance of self-management and its impact on mental health is essential to mitigating the negative effects of chronic disease on a patient’s quality of life.

## 1. Introduction

Each year, 17 million people die prematurely from chronic disease before the age of 70. Cardiovascular diseases are the leading cause, responsible for 17.9 million deaths annually, followed by cancer (9.3 million), chronic respiratory diseases (4.1 million), and diabetes (2 million, including kidney-related deaths) [1]. These four groups account for over 80% of premature chronic disease-related deaths. Risk factors such as smoking, physical inactivity, harmful alcohol use, unhealthy diets, and air pollution increase mortality risk and adversely affect mental health and well-being. Effective responses include early detection, screening, treatment, and palliative care [2,3,4]. Chronic disease represents a significant global public health challenge. These conditions are typically long-lasting and contribute to reduced quality of life and elevated mortality rates [1,3]. Effective management requires a comprehensive approach that addresses physical health and psychological, social, and general dimensions of well-being [5,6]. Mental distress can accelerate the progression of chronic disease, while the physical burden of these conditions often leads to psychological challenges [7,8]. Consequently, the primary goal in managing chronic disease should focus on enhancing quality of life rather than merely prolonging life expectancy [9,10].

The ageing process and chronic diseases reduce the body’s biological reserves, making individuals more vulnerable to health problems. This vulnerability, known as frailty, is linked to a decline in the body’s ability to function and a breakdown of key physiological mechanisms [11]. Frailty is a clinically recognisable state of increased vulnerability resulting from age-related decline in physiological reserves and functions across multiple organ systems. This condition reduces the ability to cope with every day or acute stressors, leading to a higher risk of adverse health outcomes, such as falls, hospitalisation, disability, and mortality [12]. Although frailty is not a normal aspect of primary ageing, it is often overlooked, but it is influenced by age, gender, economic status, living environment, and chronic conditions [11,13]. In older adults, frailty and loneliness often overlap, as social and emotional isolation can exacerbate physical vulnerability, creating a cycle that negatively impacts overall health and well-being [14]. Loneliness is described as feeling isolated and includes social, emotional, and existential dimensions [15] and is influenced by gender, level of education, relationship status, and contacts with friends and relatives [16]. Social isolation and loneliness adversely affect mental health [17,18], and they are associated also with poor health conditions [19,20].

Chronic disease has a significant impact on mental health, a person’s identity, and self-esteem. Chronic conditions, loneliness, frailty, stress, immune dysregulation, and mental health are intricately interconnected [21]. The persistent health challenges associated with chronic disease adversely affect both physical and psychological well-being [22]. The risk of mental health issues rises with increasing medical comorbidities. Addressing complications can enhance the quality of life for patients with chronic conditions. Effective management of such conditions necessitates better integrating mental health, primary, and speciality care, with consistent attention to patients’ emotional well-being [7,23]. Patients with conditions such as diabetes, cardiovascular disease, cancer, or respiratory disease are at increased risk of developing mental disorders [24,25]. Chronic disease often leads to emotional challenges like depression, anxiety, irritability, and fear, driven by the stress and uncertainty of managing long-term conditions [22]. At the same time, unhealthy behavioural patterns, medication non-adherence, and weakened immune systems contribute to mental health problems [23]. Mental health is a vital component of overall health, encompassing an individual’s emotional, psychological, and social well-being [26].

Self-management of chronic disease refers to the individual’s ability to actively participate in managing their health conditions by understanding their disease, adhering to treatment regimens, monitoring symptoms, and making necessary lifestyle adjustments. It involves problem solving, decision making, and engaging healthcare providers to maintain optimal health and prevent complications. Effective self-management can improve quality of life and reduce healthcare utilisation [27]. Effective self-management of chronic disease is crucial for enhancing health behaviours, outcomes, and quality of life while reducing healthcare utilisation and societal costs [28]. Supporting self-management is integral to quality care for chronic conditions. Patients with chronic conditions and their families and carers manage daily decisions impacting health and self-care. Support, guidance, and encouragement from healthcare professionals are essential to build the knowledge, confidence, and skills for effective management. These patients account for approximately 70% of healthcare resource use [6]. Because of that, patients with chronic diseases need to manage their health and care actively [29]. Self-management interventions prove more effective than routine care in managing chronic disease, significantly improving quality of life, self-efficacy, and mental health. They assist patients in adapting to disease-related changes and other challenges, successfully managing their condition, and preventing disease progression [4].

A review of existing studies shows that loneliness, frailty and mental health are related to chronic disease. At the same time, self-management is critical to the daily lives of patients with chronic diseases. There is a lack of research that considers loneliness, frailty and mental health in relation to the self-management of patients with chronic disease. A deeper understanding of the relationships between the above conditions could significantly contribute to better well-being and health and, thus, a higher quality of life for patients with chronic disease. Therefore, we wanted to investigate the relationship between loneliness, frailty, mental health, and patient self-management. In addition, the study will examine gender differences, compare marital status, and examine the role of living environment in loneliness, frailty, and mental health. These factors are crucial to understanding the nuanced experiences of patients with chronic disease and tailoring interventions to their specific needs. Research into the living environment is particularly important as people living in care homes may experience different social dynamics, access to healthcare, and physical challenges compared to those living in the community.

## 2. Materials and Methods

### 2.1. Design

This quantitative cross-sectional study analysed loneliness, frailty, and mental health in patients with one or more chronic diseases. The study strictly adhered to the STROBE (Strengthening the Reporting of Observational Studies in Epidemiology) checklist to ensure the reliability and transparency of the results.

### 2.2. Study Sites/Settings and Populations

A purposive sampling technique was used to ensure that only participants who met certain criteria relevant to the research objectives were included. This approach was chosen because of the desire to focus on adult patients with chronic conditions who could provide reliable self-reported information. To minimise sampling and recruitment bias, an attempt was made to recruit participants from different healthcare facilities in various geographical locations, including primary care centres, outpatient clinics, and nursing homes. This strategy aimed to obtain a representative sample of the wider population of patients with chronic disease in Slovenia.

The study included adult patients aged 21 years and older. This age limit was chosen to focus on people who are generally able to give informed consent and self-report their experiences of chronic disease, loneliness, frailty, and mental health. It also reflects the age at which chronic disease typically begins to impact psychosocial factors such as loneliness and mental health significantly. By excluding individuals under the age of 21, confounding factors related to developmental or adolescent-specific problems that fell outside the scope of this study were avoided. Patients with a known diagnosis of a mental disorder were excluded to reduce potential confounding factors that could influence the variables of the study.

The study was aimed at adult patients diagnosed with one or more chronic diseases. Chronicity was defined as a condition requiring constant medical care or limiting activities of daily living for six months or longer. This criterion is consistent with widely accepted definitions of chronic disease in clinical and research contexts. Eligible participants were identified by healthcare professionals in collaborating facilities, including primary care centres, outpatient clinics, and nursing homes. These professionals verified the duration of the disease using medical records or patient self-reports.

The required sample size was determined using the Cochran formula based on an estimated population of 445,454 adults with chronic disease in Slovenia. A minimum sample size of 392 (*Z* = 1.96; *p* = 0.5; *e* = 0.05) participants was calculated to achieve statistically robust results. To ensure an adequate response rate, 1100 questionnaires were distributed in various healthcare facilities. A total of 605 completed questionnaires were returned, corresponding to a response rate of 55, exceeding the required sample size and increasing the reliability of the study results.

### 2.3. Sample Size and Sampling Technique

Participants in this study completed a questionnaire, including the UCLA Loneliness scale [30], The General Health Questionnaire [31], and the Tilburg Frailty Indicator [32], and questions about self-management, contact with relatives, and other demographic questions. Chronic disease self-management was assessed using a single-item question: “How would you rate your ability to self-manage your chronic disease?” Participants responded on a 4-point Likert scale ranging from 1 (very poor) to 4 (very good). The Cronbach alpha for the questionnaire was 0.789.

The UCLA Loneliness Scale is a 20-item instrument for assessing subjective feelings of loneliness and social isolation. Participants rate each item on a 4-point Likert scale, ranging from 1 (Never) to 4 (Often). Example item: “How often do you feel left out?” The total score ranges from 20 to 80. Based on the thresholds proposed by Cacioppo and Patrick [30], the severity of loneliness was categorised as follows: a total score of <28 indicates no or low loneliness, scores between 28 and 43 represent moderate loneliness, and scores >43 indicate severe loneliness. The scale showed good internal consistency with a Cronbach’s α coefficient of 0.786.

The General Health Questionnaire (GHQ-12), developed by Goldberg and Williams [31], was used to measure mental health. This 12-item questionnaire assesses the severity of mental health problems in recent weeks using a 4-point Likert scale, ranging from 0 (Never) to 3 (Always). Example item: Have you recently been able to enjoy your day-to-day activities?” The total score ranges from 0 to 36, with lower scores indicating better mental health. Cronbach’s α for the GHQ-12 was 0.803.

The Tilburg Frailty Indicator (TFI) assesses frailty using 15 items from the three physical, psychological, and social domains. Physical frailty is assessed with eight items (Example: “Do you experience problems with walking?”), psychological frailty with four items (Example: “Do you often feel downhearted or blue?”), and social frailty with three items (Example: “Do you miss having people around you?”). The scores of all items are summarised to give a total frailty score ranging from 0 to 15, with individuals scoring ≥5 classified as frail [32]. Cronbach’s α for the TFI was 0.779.

### 2.4. Data Collection

Data were collected between October 2023 and May 2024 from patients with chronic diseases living in-home or nursing home settings after ethical consent was given and signed by participating facilities and individuals in the communities. Healthcare organisations that facilitated contact between researchers and participants facilitated recruitment. These facilities included primary care centres, outpatient clinics, and nursing homes. The healthcare professionals at these institutions identified eligible patients based on the study’s inclusion criteria and informed them of the purpose of the study. Patients who expressed interest received a questionnaire and instructions. To encourage participation in the study, the questionnaires were distributed in person by the research team, who were available to clarify any doubts about the study or its objectives. Participants returned the completed questionnaires in sealed envelopes to secure facility collection centres within seven days. This approach ensured that the sample included patients with different living environments and access to healthcare, which was essential to capture the broad dynamics of chronic disease, loneliness, frailty, and mental health.

During the data analysis, all returned questionnaires were checked for completeness. Cases with missing data on critical variables (e.g., loneliness, frailty or mental health scales) were excluded from certain analyses to maintain the validity of the results. For minor gaps in non-critical demographic data, pairwise deletion was undertaken to maximise the use of available data without introducing bias. A total of 605 completed questionnaires were included in the final analysis, representing a response rate of 55%.

### 2.5. Data Analysis

This study utilised both descriptive and inferential statistical methods. The data were analysed using SPSS Statistics 25.0. The Shapiro–Wilk test assessed the normality of variable distributions, revealing that the data deviated from a normal distribution. Accordingly, non-parametric tests, such as the Kruskal–Wallis and Mann–Whitney U-tests, were used to compare group differences. Spearman’s correlation coefficient was applied to the relationships between loneliness, frailty, mental health, and self-management. A two-way ANOVA was conducted to examine the interactions between gender and living environment on frailty, loneliness, and mental health. The interaction effects were significant for loneliness (F(1, 603) = 4.15, *p* = 0.042, η^2^ = 0.02). A Bonferroni correction was applied to account for multiple comparisons and to ensure a conservative approach to identifying significant differences. Effect sizes, including Cohen’s d and η^2^, were calculated for all significant results to provide insight into the magnitude of differences. A significance of *p* < 0.05 was set for all statistical analyses. The internal consistency of the questionnaire was assessed using Cronbach’s α coefficient.

### 2.6. Ethical Considerations

Formal approval was obtained from the relevant ethical committee (Ref. No: 038/2022/5006-4/902) before initiating the study. Permissions were also secured from retirement homes and individual participants. Ethical guidelines were followed, including providing participants with all necessary information on the first page of the questionnaire. The researchers strictly adhered to the principles of the Declaration of Helsinki and the provisions of the Oviedo Convention, ensuring the protection of patients’ rights, adequate information, and voluntary participation and maintaining the integrity of the research process.

## 3. Results

In total, 605 patients with chronic disease participated in the study; 404 (67%) were female, while 201 (33%) were male. In total, 287 (47%) patients were older than 65, and 156 (54%) lived in retirement homes. In total, 432 (71%) community-dwelling patients with at least one chronic disease and 173 (29%) patients with at least one chronic disease who lived in retirement homes participated in the research. Patient demographics are summarised in Table 1.

The average age of the participating patients was 59 years (*SD* = 17.04, Min:24, Max:96; IC = 57.64–60.22; *Me* = 63; IQR = 26), 42% were married, and 19% were widowed. More than half of the patients (55%) had at least a secondary level of education. In total, 58% of the participating patients were multimorbid. In total, 71% self-assessed that they are managing their chronic disease well or very well.

An average score of the assessment of participating patients’ level of loneliness was 38.28 (*SD* = 10.4; Min = 20 Max = 58; 95%; IC = 37.47–39.11; *Me* = 37.0; IQR = 19.0). When we used cut-off points for the level of loneliness, we found that 116 (19%) had no or low level of loneliness, 263 (43%) had moderate loneliness, and 228 (38%) had a high level of loneliness. Of those who are older than 65 years, 45% had a high level of loneliness, and in a group younger than 65 years, 35% were those with a high level of loneliness. Of community-dwelling patients, 21% had no or low loneliness, and 31% had high loneliness, but in retirement homes, 8.1% had no or low loneliness, and 57% had high loneliness. In loneliness, there was a significant difference among patients from retirement homes (42.79 ± 10.63) and community-dwelling patients (36.47 ± 9.7) (*Z* = −6.872; *p* < 0.001). The total score for frailty was 6.53 (*SD* = 3.79; Min = 0; Max = 14; 95%; IC = 6.25–6.83; *Me* = 6.0; IQR = 6.0). According to the cut-off points (5 or more), 64% of participating patients are frail; of those in the communities, 55%, and in retirement homes, 90%. When we compared those younger or older than 65, we found that in a group 65 or older, 75% of participating patients were frail. In a group younger than 65, 39% were frail. The total score of frailty was higher *Z* = −10.952; *p* < 0.001) from participating patients from retirement homes (9.2 ± 3.2) than in the community (5.5 ± 3.5). The score for physical frailty was 3.51 (*SD* = 2.67; Min = 0 Max = 8; 95%; IC = 3.3–6.8; *Me* = 3.0; IQR = 5.0), for psychological frailty was 1.96 (*SD* = 1.26; Min = 0, Max = 4; 95%; IC = 1.86–2.1; *Me* = 2.0; IQR = 2.0), and for social frailty was 1.06 (*SD* = 0.73; Min = 0, Max = 3; 95%; IC = 0.99–1.11; *Me* = 1.0; IQR = 0.0). The total score for mental health was 5.48 (*SD* = 1.92; Min = 0, Max = 16; 95%; IC = 17.25–17.71; *Me* = 6.0; IQR = 1.0). Community-dwelling patients assess their mental health (5.11 ± 1.96) a little better (*Z* = −5.064; *p* < 0.001) than patients from retirement homes (6.4 ± 1.59).

From Table 2, we can see that only in the level of frailty we find a significant difference according to gender (U = 36305.0; *p* = 0.033). According to age, we find significant differences (*p* < 0.001) in loneliness, frailty, and mental health. In relationship status, we found substantial differences in loneliness (H= 82.495; *p* < 0.001), frailty (H = 98.467; *p* < 0.001), and mental health (H = 45.293; *p* < 0.001). Also, according to the level of education, there were significant differences in loneliness (H = 16.084; *p* < 0.001), frailty (H = 50.897; *p* < 0.001), and mental health (H = 49.052; *p* < 0.001). Significant differences were found in loneliness (H = 31.233; *p* < 0.001), frailty (H = 106.981; *p* < 0.001), and mental health (H = 10.533; *p* = 0.015) according to managing chronic disease. According to the number of contacts with relatives, there are significant differences in loneliness (H = 21.873; *p* < 0.001) and frailty (H = 22.144; *p* < 0.001). Effect sizes were calculated for all significant comparisons, with Cohen’s d indicating moderate effects for differences in frailty between living environments (*d* = 0.62) and loneliness between marital statuses (*d* = 0.54).

Table 3 shows loneliness, frailty, and mental health among patients with at least one chronic disease in different living environments.

Table 3 shows that according to gender, loneliness was significantly different in retirement homes (U = 8.418; *p* = 0.039) and communities (U2.526; *p* = 0.012). Research showed significant differences in the level of frailty, loneliness, and mental health according to age among patients in retirement homes and community dwellings. Significant differences in loneliness were also found according to relationship status in retirement homes (U = 8.031; *p* = 0.045) and in the community (U = 13.785, *p* = 0.028). According to relationship status, there was a difference in mental health but only among patients from communities (U = 7.849; *p* = 0.049). Widowed patients were more lonely and frailer and had poorer mental health than married and cohabited. There was a significant difference in frailty according to education level in retirement homes (U = 9.779; *p* = 0.021) and community dwellings (U = 30.338; *p* < 0.001). There were no significant differences in studied variables according to the number of chronic diseases. According to self-management of chronic disease, significant differences were found in loneliness (*p* < 0.05), frailty (*p* < 0.001), and mental health (*p* < 0.001) in retirement homes and community dwellings.

We found that those patients who have contacts more often are less frail (*r_s_* = 0.121; *p*= 0.003) and less lonely *(r_s_* = 0.186; *p* < 0.001). It was also found that patients who assessed self-managing chronic disease were less frail (*r_s_* = 0.356; *p* < 0.001) and had better mental health (*r_s_* = 0.146; *p* < 0.001). It was also found that frailty is associated with loneliness *(r_s_* = 0.428; *p* < 0.001) and lower mental health *(r_s_* = 0.185; *p* < 0.001), and that loneliness is associated with lower mental health *(r_s_* = 0.099; *p* = 0.014) (Table 4).

A partial correlation analysis analysed the relationships between loneliness, frailty, mental health, and various control variables. Controlling for age, there was a moderate, positive correlation between loneliness and frailty (*r* = 0.398, *n* = 424, *p* < 0.001) and a weak to moderate, negative correlation between loneliness and mental health (r = −0.269, *n* = 424, *p* < 0.001). Frailty and mental health also showed a weak to moderate negative correlation (r = −0.240, *n* = 424, *p* < 0.001) when controlling for age. These correlations suggest that age has a modest moderating effect, as the zero-order correlations showed slightly stronger correlations. Loneliness and self-management showed no significant correlation (r = 0.002, *n* = 424, *p* = 0.970), both with and without controlling for age. A weak positive correlation was found between loneliness and place of residence when controlling for age (r = 0.217, *n* = 424, *p* < 0.001), with a stronger correlation on a null basis (r = 0.441, *n* = 424, *p* < 0.001), suggesting that age moderates this relationship. Finally, the frequency of contact with friends or relatives was weakly positively associated with loneliness (r = 0.131, *n* = 424, *p* = 0.007), with age having a minimal moderating effect, as evidenced by a stronger zero-order correlation (r = 0.441, *n* = 424, *p* < 0.001). Overall, the results show that although age plays a role in moderating some relationships, the most important associations remain significant even after accounting for this influence.

With the regression analysis, we found that loneliness (β = 0.152, t = −3.552, *p* < 0.001) and frailty (β = −0.337, t = 8.769, *p* < 0.001) can explain 39% of the total variability of self-management of chronic disease. When we added economic status, relationship status, age, and number of chronic diseases to the model, we saw that we could explain 47.8% of the total variability of chronic disease self-management. From all dependent variables, only the number of chronic diseases did not have a significant effect (*p* = 0.298). After, we added all three different frailty types separately into the model. We saw that only physical and social frailty had a significant effect (*p* < 0.001) on chronic disease self-management. At the same time, it was also found that with chronic disease self-management, we can explain 14% of the total variability of mental health (β = −0.140, t = 3.461, *p* < 0.001).

## 4. Discussion

In our research, we wanted to explore an association between self-management of chronic disease, frailty, loneliness, and mental health among patients with one or more chronic diseases. The study’s main findings are that the participating patients with chronic disease had good mental health, and more than 82% rated their chronic disease self-management as good or very good. Still, more than 40% are moderately lonely, and more than 60% are frail. The results suggest that self-management is related to frailty, loneliness, and mental health. Patients who rate their self-management as good or very good are less frail and less lonely, and their mental health is better. Some demographic characteristics such as education level, number of chronic diseases, age, relationship status, and level of self-management were identified as factors influencing loneliness, frailty, and mental health.

Additional moderation analyses were conducted to investigate whether the relationship between frailty and self-management depends on contextual factors such as place of residence. Interaction effects between frailty and living environment (shared flats vs. retirement homes) were tested using regression models with interaction terms. These analyses showed that the association between frailty and self-management was stronger for people living in retirement homes (β = −0.45, *p* < 0.001) than for people living in shared housing (β = −0.27, *p* = 0.018). This result suggests that the living environment moderates the impact of frailty on self-management, with nursing home residents possibly facing additional barriers, such as reduced autonomy or limited access to resources, which exacerbate the effects of frailty on their ability to self-manage.

Our research shows that of all participating patients with chronic disease, 43% are moderately lonely, and 38% are severely lonely. Of the older adult patients, 45% are severely lonely, and 43% are moderately lonely. Of the participating patients in nursing homes, 57% were severely lonely, and 35% were moderately lonely. Compared to the results in older adults living in nursing homes, Hayek et al. [33] found that approximately 56.6% of individuals reported no loneliness, 25.7% were moderately lonely, and 17.8% were severely lonely. The regression analyses revealed that higher levels of loneliness were associated with being married, having a higher level of education than those with a lower level of education, having a smaller social network, having poorer health, and having a greater number of depressive symptoms [33]. In comparison, our study also found that people with a lower level of education, who are older, who have less contact with relatives, who are divorced, widowed, and single have higher levels of loneliness.

The study [34], which covered 27 European countries, also found that loneliness tended to increase with increasing age, transition from marriage and cohabitation with a spouse/registered partner to another marital status, reduction in logbook entry, deterioration in self-rated health, deterioration in functional status, increase in depressive symptoms, and impairment in cognitive functioning. However, changes in the presence of chronic disease were not associated with loneliness [34]. Although the number of chronic diseases was considered a potential moderating variable, no significant interaction effects were observed between the number of chronic diseases and self-management concerning frailty (interaction term *p* = 0.256). This finding suggests that while the overall burden of chronic disease influences self-management, its effect is likely mediated by other factors such as symptom severity, support systems, or access to care and is not a direct moderator. The data show the same results regardless of whether they live in shared housing or retirement homes. The only significant difference between living environments was loneliness according to gender, where we found that women were lonelier than men. Other studies in the general population and during the COVID-19 pandemic also found that more women are lonely than men [16,35,36].

The data show that 64% of all participating patients with chronic diseases are frail. By age (up to 65 years), 49% were frail, and 82% were frail in the older adult group. Regarding the living environment, 55% of patients in the community and 90% in nursing homes were frail. Our findings are supported by those of Hladek et al. [37], who found that half of community-dwelling participants with at least one chronic disease were categorised as frail or prefrail. They [37] found that after adjusting for age, gender, ethnicity, comorbidities, heart rate, number of life events, and body mass index, high coping self-efficacy was associated with a 92% reduction in the odds of being frail. Our research found that physical frailty was the highest regardless of the living environment, followed by psychological and social frailty. Physical and psychological frailty predict mortality [38], which is important for healthcare workers and society.

The data also show that patients with chronic diseases with frequent contact with relatives are less frail and less lonely. It was also found that patients who are better able to self-manage their chronic disease are less frail and have better mental health. At the same time, frailty was found to be associated with loneliness and lower mental health, and loneliness was also associated with lower mental health. Our results are supported by the findings of Mantell et al. [39], who found that psychosocial and structural factors, including loneliness, residence in an impoverished neighbourhood, and being female, were significantly associated with frailty and functional decline. In our study, the participating patients with chronic diseases had good mental health (M = 5.84 (Me = 6) out of 36), but there is still a significant difference in education level, relationship status, and self-management. It was also found that patients with chronic diseases living in the community had better mental health than patients in nursing homes.

The data also show that 82% of the participating patients with chronic diseases rated their self-management as good or very good. Patients living in a community rated their self-management as good or very good in 92, and 48% of patients from nursing homes. There were significant differences in loneliness, frailty, and mental health depending on the level of self-management, regardless of place of residence. The importance of self-management supports the findings of a systematic review and meta-analysis [4] of 7603, including patients with chronic diseases, which concluded that self-management interventions significantly improved quality of life and self-efficacy and reduced symptoms of depression compared to usual care.

Finally, we also found that loneliness and frailty can explain 39% of the overall variability in patients’ self-management of chronic diseases. Loneliness and frailty, together with economic status, relationship status, education level, age, and number of chronic diseases, can explain 48% of the total variability in patients’ chronic disease self-management. Of all the dependent variables, only the number of chronic diseases had no significant effect on self-management. At the same time, it was also found that we can explain 14% of the total variability of mental health with chronic disease self-management. Still, there are also a lot of other known variables that influence mental health but are not considered in the research. The following results [40,41] support our findings. In a fixed-effects model that adjusted for marital status, age, gender, wealth, and smoking status, higher levels of loneliness and social isolation were found to be associated with higher levels of frailty. In addition, older age was associated with greater frailty, independent of loneliness or social isolation. Both loneliness and social isolation increase the risk of frailty [40]. A systematic review and meta-analysis also confirmed that greater frailty is strongly associated with greater loneliness [41]. Based on our findings, we agree that there may be a bidirectional relationship between loneliness and frailty in older adults. We also agree that older adults who suffer from loneliness should be made aware of the potentially self-reinforcing cycle of loneliness and its impact on their health [42].

Patients with chronic diseases often struggle with persistent health problems that not only lead to negative health outcomes but also affect their mental health and drive up healthcare costs. Therefore, healthcare professionals must develop successful management plans to help these patients manage their conditions and improve their mental health. This study highlights the impact of loneliness and frailty on chronic disease self-management and emphasises the need for continuous monitoring to enable early detection. We agree with Budreviciute et al. [43], who found that various interventions can help patients prevent frailty and loneliness, adapt to the changes caused by their disease, and manage their disease effectively to slow progression. In addition, self-management interventions play a crucial role in improving mental health and optimising health resources by enabling patients to adopt healthier behaviours and develop skills for better self-management of chronic conditions. This, in turn, improves patient outcomes and reduces the overall disease burden.

Several limitations of this study merit consideration. Cross-sectional design limits the ability to establish causal relationships. Only a few published studies are comparing the loneliness, frailty, and mental health of patients with chronic disease, those living in communities, and those living in retirement homes. Secondly, cross-sectional studies are susceptible to recall bias. Another potential limitation is the sampling method and its influence on the findings. Due to the overrepresentation of one gender and location in the sample, the results may not be generalizable to the broader population. Additionally, the reliance on self-reported data may have introduced social desirability bias, as participants might have exaggerated or downplayed their responses to align with societal expectations. Moreover, the voluntary nature of participation raises concerns about the sample’s representativeness regarding the key characteristics of the population. Additionally, not all potential confounding factors that could impact self-management, loneliness, frailty, and mental health could be accounted for in this study.

Despite some limitations, this study emphasised the critical importance of early detection of loneliness and frailty to enhance self-management and inform initial healthcare strategies to support patient’s self-management of chronic disease, which significantly impacts mental health. Early recognition of loneliness and frailty significantly improves chronic disease self-management by identifying individuals at risk and enabling timely interventions [13,44]. This is particularly important given the increasing number of older adults and the growing prevalence of chronic disease [1,43,44]. Equally important is the realisation that every person with a chronic disease, regardless of age, education level, or disease type, must actively self-manage their health.

Addressing these issues can prevent the exacerbation of chronic conditions, reduce healthcare costs and improve overall quality of life. However, there are still gaps in routine screening, awareness raising, and integration of psychosocial factors into chronic disease management protocols. We have put together some practice recommendations that, if applied systematically, can close the gaps in screening and empower individuals to manage their health proactively:

Early detection of frailty and loneliness should be prioritised in primary care and communities. This can be achieved by introducing regular screening programmes using standardised instruments such as the Tilburg Frailty Indicator and the UCLA Loneliness Scale. These tools have been validated in different populations and can facilitate the identification of at-risk individuals, allowing for timely interventions. Recent studies have shown that early intervention significantly improves health outcomes and reduces the progression of frailty and loneliness.

Interdisciplinary collaboration is essential to meet the complex needs of patients with chronic diseases. Partnerships between healthcare providers, social workers, and community organisations should be strengthened to develop comprehensive care plans that integrate medical and psychosocial support. Research shows that such integrative approaches can improve quality of life, promote disease self-management, and reduce healthcare utilisation.

Digital health technologies hold great promise for monitoring and managing frailty and loneliness. Wearable devices and mobile applications can track mobility, physical activity, and social interactions and provide real-time data to healthcare providers. These technologies have increased patient engagement and adherence to treatment plans, ultimately contributing to better health outcomes.

Community-based interventions should be expanded to promote social engagement and reduce loneliness. Initiatives such as group exercise classes, peer support groups, and volunteer networks effectively promote social contact and mitigate the negative effects of isolation. Programmes for older adults and people in residential care are particularly effective given their increased vulnerability.

Patient education and empowerment must remain central to chronic disease management strategies. Providing resources, education, and support for patients to build self-management skills can improve resilience and functional independence. There is evidence that self-management interventions significantly improve self-efficacy, mental health, and quality of life while reducing symptoms of depression and healthcare costs.

Finally, political lobbying is crucial for the institutionalisation of these recommendations. Governments and healthcare systems should adopt guidelines that integrate the assessment of loneliness and frailty into the routine management of chronic diseases. Successfully implementing these measures requires adequate funding and training of healthcare professionals. Promoting a supportive policy environment can significantly improve the long-term well-being of people with chronic diseases.

Our findings also emphasise the need for a nuanced approach when investigating predictors of self-management, particularly in vulnerable subgroups. Future research should utilise advanced statistical techniques such as structural equation modelling or hierarchical linear models to capture complex interactions between variables, including social support, economic status, and environmental factors. In addition, longitudinal studies could further clarify causal pathways and identify critical points for intervention.

## 5. Conclusions

The rising proportion of older adults, along with the growing burden of chronic diseases and the increased vulnerability of this population, highlights the pressing need to transform healthcare strategies and encourage preventive approaches to ensure healthy ageing.

For individuals with chronic diseases to take responsibility and successfully manage their condition, they must be empowered to make informed and responsible decisions about their health. Without proper assessment and support from the healthcare system, patients may not struggle to meet their needs, potentially exacerbating issues such as loneliness, frailty, and mental health decline. The primary goal of public health is to promote independence in daily life and enhance overall mental health and well-being.

Gaining insight into how social, environmental, and biological factors contribute to frailty and loneliness, along with regular monitoring, can help mitigate its adverse health effects and support the development of effective healthcare strategies for early and old life. Only understanding the importance of a patient’s self-management of chronic disease and its effects on mental health can help to prevent the negative impact of chronic disease on a patient’s quality of life.

## Figures and Tables

**Table 1:** Demographic characteristics.

Variables	Descriptive Statistics Total (*n* = 605)
Gender	% (n)
Female	66.8 (404)
Male	33.2 (201)
Relationship Status	% (n)
Single	10.2 (62)
Married	44.3 (268)
Divorced	7.8 (47)
Cohabitation	19 (115)
Widowed	18.7 (113)
Level of education	% (n)
Elementary education	11.2 (68)
Secondary Education	54.7 (331)
HE (Bachelor)	26.8 (162)
HE (Master or Doctoral)	7.3 (44)
Number of chronic diseases	% (n)
One	41.7 (253)
Two to three	40.0 (242)
Four or more	18.2 (110)
Self-management of chronic disease	%(n)
Very poor	7.9 (45)
Poor	11.4 (69)
Well	73.2 (443)
Very well	7.4 (48)

**Table 2:** Relationship of loneliness, frailty, and mental health according to the demographic characteristics.

**Variables**	**Loneliness**	**Frailty**	**Mental health**
	M ± SD	M ± SD	M ± SD
	(Me ± IQR)	(Me ± IQR)	(Me ± IQR)
	38.28 ± 10.37	6.53 ± 3.79	5.48 ± 1.92
	(37.0 ± 19.0)	(6.0 ± 6.0)	(6.0 ± 2.0)
Gender	U = 38,072.0	U = 36,305.0	U = 19,291.5
	*p* = 0.211	*p* = 0.033	*p* = 0.289
Male	38.94 ± 9.79	6.07 ± 3.74	5.79 ± 1.08
	(37.0 ± 20.0)	(6.0 ± 5.50)	(18.0 ± 1.0)
Female	37.95 ± 10.64	6.76 ± 3.79	5.33 ± 1.83
	(36.0 ± 19.0)	(6.0 ± 6.0)	(18.0 ± 1.75)
Age	*r_s_* = 0.277	*r_s_* = 0.487	*r_s_* = 209
	*p* < 0.001	*p* < 0.001	*p* < 0.001
Relationship Status	H(4) = 82.495	H(4) = 98.467	H(4) = 45.293
	*p* < 0.001	*p* < 0.001	*p* < 0.001
Single	38.48 ± 9.99	6.27 ± 3.82	3.90 ± 1.58
	(39.0 ± 19.0)	(5.0 ± 7.0)	(5.0 ± 1.25)
Married	36.31 ± 9.94	5.57 ± 3.52	5.41 ± 1.81
	(33.0 ± 19.0)	(5.0 ± 4.0)	(6.0 ± 0.50)
Divorced	39.55 ± 13.07	7.85 ± 3.15	7.13 ± 1.53
	(45.0 ± 30.0)	(9.0 ± 6.0)	6.0 ± 2.0
Cohabitation	34.72 ± 8.18	5.37 ± 3.02	4.75 ± 1.67
	(33.0 ± 10.0)	(5.0 ± 5.0)	(18.0 ± 2.0)
Widowed	45.93 ± 8.32	9.58 ± 3.56	6.08 ± 1.84
	(49.0 ± 8.0)	(11.0 ± 6.0)	(18.0 ± 2.0)
Level of education	H(3) = 16.084	H(3) = 50.897	H(3) = 49.052
	*p* < 0.001	*p* < 0.001	*p* < 0.001
Elementary education	40.16 ± 10.23	7.0 ± 3.61	6.07 ± 2+1.21
	(45.5 ± 20.0)	(6.5 ± 6.0)	(6.0 ± 1.75)
Secondary Education	39.27 ± 10.42	7.35 ± 3.81	5.75 ± 1.03
	(39.0 ± 20.0)	(7.0 ± 7.0)	(6.0 ± 2.0)
HE (Bachelor)	36.14 ± 10.54	5.31 ± 3.45	5.10 ± 1.53
	(34.0 ± 20.0)	(5.0 ± 5.0)	(6.0 ± 2.0)
HE (Master or Doctoral)	35.14 ± 7.40	4.14 ± 2.83	3.93 ± 1.69
	(34.0 ± 13.0)	(4.0 ± 3.0)	(6.0 ± 2.0)
Number of chronic diseases	H(1) = 2.963	H(1) = 1.393	H(1) = 0.857
	*p* = 0.085	*p* = 0.238	*p* = 0.355
One	37.49 ± 10.48	6.25 ± 3.87	5.38 ± 2.93
	(38.0 ± 19.0)	(6.0 ± 6.0)	(6.0 ± 2.0)
Two to three	38.75 ± 10.09	6.86 ± 3.50	5.50 ± 1.83
	(35.0 ± 20.0)	(6.0 ± 5.0)	(6.0 ± 1.0)
Four or more	41.0 ± 12.3	8.1 ± 6.49	4.4 ± 2.03
	(50.0 ± 26.0)	(11.0 ± 14.0)	(6.0 ± 2.25)
Self-management of chronic disease	H(3) = 31.233	H(3) = 106.981	H(3) = 10.533
	*p* < 0.001	*p* < 0.001	*p* = 0.015
Very poor	44.73 ± 10.45	7.75 ± 2.98	6.15 ± 0.83
	(44.5 ± 19.75)	(8.0 ± 5.75)	(6.0 ± 0.0)
Poor	44.28 ± 9.43	10.87 ± 2.81	6.15 ± 0.93
	(48.0 ± 12.0)	(12.0 ± 2.50)	(6.0 ± 1.0)
Well	37.85 ± 10.4	5.89 ± 3.58	5.44 ± 2.16
	(36.0 ± 20.0)	(6.0 ± 5.0)	(6.0 ± 1.0)
Very well	37.09 ± 7.79	4.88 ± 2.83	4.18 ± 1.47
	(34.0 ± 14.0)	(5.0 ± 4.0)	(6.0 ± 2.0)
Frequency of contact with relatives	H(3) = 21.873	H(3) = 22.144	H(3) = 1.632
	*p* < 0.001	*p* < 0.001	*p* = 0.652
Less than once/month	40.07 ± 9.06	7.32 ± 3.72	5.59 ± 1.72
	(42.0 ± 18.5)	(8.0 ± 7.0)	(6.0 ± 0.0)
Once/week to once/month	40.68 ± 10.98	7.66 ± 3.94	5.67 ± 1.56
	(44.0 ± 19.5)	(8.0 ± 7.0)	(6.0 ± 1.0)
Few timed/week	38.84 ± 10.58	5.80 ± 3.98	5.49 ± 2.08
	(37.5 ± 21.0)	(5.0 ± 5.0)	(6.0 ± 2.0)
Daily	36.16 ± 9.66	6.24 ± 3.39	5.41 ± 1.53
	(34.0 ± 20.0)	(6.0 ± 4.0)	(6.0 ± 1.0)

Note: *M*—Mean; *SD*—Standard Deviation; *Me*—Median; IQR—Interquartile Range; *p*-value—statistically significant (<0.05); r_s_ coefficient—Spearman’s Rank Correlation Coefficient; H coefficient—Kruskal–Wallis H Test Statistic; U coefficient—Mann–Whitney U Test Statistic.

**Table 3:** Results of loneliness, frailty, and mental health of patients in retirement homes and communities.

Variables	Loneliness		Frailty		Mental Health	
	Retirement Home	Community-Dwelling	Retirement Home	Community-Dwelling	Retirement Home	Community-Dwelling
	(*n* = 173)	(*n* = 432)	(*n* = 173)	(*n* = 432)	(*n* = 173)	(*n* = 432)
Gender	8.418	2.526	0.895	1.469	1.037	0.674
*U*; *p*	0.039	0.012	0.371	0.142	0.300	0.500
Age	0.296	0.130	0.405	0.437	0.128	0.147
*r_s_*; *p*	<0.001	0.007	<0.001	<0.001	0.010	0.002
Relationship Status	8.031	13.785	2.186	3.983	3.192	7.849
H(3); *p*	0.045	0.028	0.535	0.263	0.363	0.049
Level of Education	28.383	5.882	9.779	30.338	6.667	45.847
*H*(3); *p*	<0.001	0.118	0.021	<0.001	0.083	<0.001
Number of chronic diseases	0.552	0.924	0.020	0.123	0.341	0.854
H(1); *p*	0.470	0.337	0.889	0.726	0.154	0.695
Self-management of chronic disease	32.126	11.138	21.918	36.765	18.716	32.760
*H*(3); *p*	<0.001	0.011	<0.001	<0.001	<0.001	<0.001
Mann–Whitney U test	24,029		16,159		11,028	
	<0.001		<0.001		<0.001	

Note: *p*-value—statistically significant (<0.05); r_s_ coefficient—Spearman’s Rank Correlation Coefficient; H coefficient—Kruskal–Wallis H Test Statistic; U coefficient—Mann–Whitney U Test Statistic; F—F value is the ratio of the Between and Within variation.

**Table 4:** Results of Spearman correlations for frailty, loneliness and mental health.

Variables	Frequency of Contacts	Self-Management	Frailty	Loneliness	Mental Health
Frequency of contacts	1	0.420 **	−0.121 **	−0.186 **	−0.012
Self-management	0.420 **	1	−0.356 **	0.075	−0.146 **
Frailty	0.121 **	0.356 **	1	0.428 **	0.185 **
Loneliness	0.186 **	0.075	0.428 **	1	0.099 *
Mental Health	0.012	0.146 **	0.185 **	0.099 *	1

Note: *—correlation is significant at the 0.05 level; **—correlation is significant at the 0.01 level.

## Data Availability

Data are contained within the article.
